# Proteoglycan Dynamics and Bone Quality: Molecular Regulation to Age-Related Fragility

**DOI:** 10.3390/biom16040572

**Published:** 2026-04-13

**Authors:** Savannah Heath, Rui Hua, Xiaodu Wang, Jean Jiang

**Affiliations:** 1 Biochemistry and Structural Biology Department, University of Texas Health Science Center at San Antonio, San Antonio, TX 78229, USA; heaths@uthscsa.edu (S.H.); huar@uthscsa.edu (R.H.); 2 Mechanical Engineering Department, University of Texas at San Antonio, San Antonio, TX 78249, USA; xiaodu.wang@utsa.edu

**Keywords:** glycosaminoglycans (GAGs), proteoglycans (PGs), bone quality, bone toughness, aging, fragility fracture

## Abstract

Clinically, bone mineral density (BMD) accounts for only approximately 50% of the observed variance in bone fragility fractures. This review examines the dynamic and mechanistic role of the non-collagenous organic matrix, specifically proteoglycans (PGs) and glycosaminoglycans (GAGs), in maintaining bone toughness and bone quality. During aging, bulk cortical GAG levels decrease by up to ~17% and are highly associated with reduced bone tissue toughness. We analyze how this age-related loss may arise from uncoupled bone remodeling and tissue aging, including the accumulation of older, interstitial tissue and dysregulated osteocyte-mediated matrix maintenance. We then discuss the functional importance of PG/GAG composition, maturation, and catabolism and how perturbations in these processes can promote pro-inflammatory signaling that accelerates matrix degradation and contributes to systemic aging. Lastly, we discuss potential interventions to preserve or restore GAGs/PGs in bone and improve overall bone quality.

## 1. Introduction

Humans experience an increased incidence of fragility fractures with age, which can often result in reduced quality of life, a high economic burden, elevated morbidity risks, and even premature death [1,2,3,4,5]. Additionally, the incidence of fragility fracture is significantly higher in females than males, with approximately 1/3 of women over 50 years expected to fracture at least once in their lifetime compared to ~1/5 in males [6]. The most common risk factor for fractures is bone mineral density (BMD). Clinically, BMD is obtained from a dual-energy X-ray absorptiometry (DXA) scan at common fracture locations like the hip, wrist, and vertebral bodies. BMD values can be scored against a healthy population to diagnose osteoporosis. Patients with a BMD score of 2.5 standard deviations (T score < −2.5) below the mean are osteoporotic. Osteoporosis is defined as the loss of bone mass; however, this only accounts for around 50% variance in bone fragility fractures in patients [7,8,9,10]. In fact, there are several reports demonstrating that age-related fractures are increasing independent of changes in bone mass [11,12].

Intuitively, fracture risk can be influenced by significant variables outside of BMD such as genetics, hormonal, and metabolic disease factors. Interestingly, these become more prevalent with age as a covariate. Other diagnostic tools like FRAX clinically supplement BMD scores by evaluating other factors like patient and parental history of fractures, sex, modifiable risk factors, and other pathologies [12]. Tools analyzing DXA textures like Trabecular Bone Score can further evaluate microarchitecture by providing an indirect assessment of the bone’s structural decay, while newer modalities like HR-pQCT and bone microindentation offer more direct insights into volumetric microarchitecture and tissue-level mechanical resistance [13]. These can improve diagnostic assessment for fracture risk, although they are extrinsic in nature, and still do not address the intrinsic root cause and corresponding therapeutic to treat age-related fragility fractures. Thus, it is important to assess intrinsic, age-dependent changes in the material quality of bone and how it plays a major role in fracture risk.

Proteoglycans (PGs), a type of extracellular matrix (ECM) non-collagenous component, are critical for bone toughness [14]. PGs contain glycosaminoglycans (GAGs), hydrophilic, sulfated polysaccharide chains. The decline in non-collagenous proteins, particularly PGs and their GAG chains, may contribute to age-related reductions in bone toughness, in part by the associated reduction in matrix-bound water [14]. Unfortunately, GAGs decrease by as much as 17% with aging in bulk cortical tissue [14], suggesting they may be a key player in age-related loss of bone quality. This review will focus on PGs/GAGs in bone, including their regulation and turnover, age-related changes, biomechanical and biological implications, and potential interventions to preserve or restore GAGs and improve bone quality.

## 2. PG Regulation in Bone

Within bone tissue, PGs play significant structural, cellular, regulatory, and mechanical roles that contribute to overall bone quality. The composition, abundance, and functional properties of PGs depend on multiple anabolic and catabolic factors, as well as microenvironment changes during local tissue aging and bone remodeling.

### 2.1. PG Metabolism

Before investigating age-associated loss of GAGs, it is necessary to understand the anabolic and catabolic mechanisms that regulate PG and GAG levels.

#### 2.1.1. PG Synthesis, Secretion, and Locations

PGs consist of a core protein often covalently attached with one or more GAG chains, which are long, unbranched polysaccharides containing repeating disaccharide units [15,16]. GAG chains are negatively charged and generate high fixed-charged density, promoting matrix hydration by attracting water into the bone matrix. The basic GAG repeating unit is a disaccharide consisting of an amino sugar and (in most cases) a uronic acid to yield five major types: chondroitin sulfate (CS), dermatan sulfate (DS), keratin sulfate (KS), heparin sulfate (HS), and hyaluronic acid (HA). Matrix PGs include small leucine rich PGs (SLRPs: decorin (Dcn), biglycan (Bgn), fibromodulin (FMOD)), type IX collagen-associated PGs, and large aggregating PGs (aggrecan and related lecticans such as brevican, neurocan, versican), and others (perlecan, agrin, and syndecan) [17]. Bgn and Dcn are the predominant PGs in bone, with two and one strands of CS GAGs, respectively. Both Bgn and Dcn can also carry DS chains.

The synthesis of PGs is a complex and highly regulated process. The major genes encoding enzymes and regulatory factors involved in PG synthesis and post-translational modifications are listed in Table 1. The core protein is synthesized in the rough ER and transported to the Golgi for glycosylation. Xylosyltransferases recognize Ser-Gly-X-Gly motifs on the core protein and add a xylose sugar to the serine to initiate linkage-region formation and GAG formation. A conservated linkage-region tetrasaccharide (Xylose (Xyl)-galactose (Gal)-Gal-glucuronic acid (GlcA)) is then added and is shared by most CS/DS and HS chains. Then, as described in more detail elsewhere, chain initiation (the first hexosamine addition) commits the structure towards either HS (α-GlcNAc) or CS/DS (β-GalNAc) biosynthesis [18]. Next, the backbone is elongated by sequential addition of disaccharide GAG units by specific glycosyltransferases. After polymerization, the chain is modified with sulfotransferases and epimerases, and the mature PGs are trafficked to the cell surface and/or secreted into the extracellular matrix [19,20].

PGs are primarily located in the extrafibrillar matrix (EFM) of bone [15,25,26]. More specifically they can localize at the cell surface (associated with membrane proteins and receptors), within the perilacunar–canalicular space, or throughout the bone extracellular matrix.

#### 2.1.2. Catabolic Enzymatic and Non-Enzymatic Mechanisms

The breakdown of extracellular PGs can arise from both enzymatic and non-enzymatic mechanisms that target either the core protein or its attached GAG chains. Table 2 summarizes major catabolic factors, their localization, principal substrates/target, and relative expression in osteoblasts, osteocytes, and osteoclasts.

Enzymatically, the core proteins can be digested with proteinases and cathepsin. Extracellularly, matrix metalloproteinases (MMPs) are among the most plausible mediators of core protein fragmentation in the extracellular matrix. Humans and mice express 23–25 MMPs, with differential expression depending on cell type. Most MMPs are optimally active at near-neutral pH (pH 7–7.5) and are primarily regulated via extracellular activation and inhibition by tissue inhibitors of metalloproteinases (TIMPs). Furthermore, experimental results have found that MMP, -3, -9, -12, -13, and -14 as well as ADAMTS-5 and -4 can cleave Bgn [33,34,35,36], while MMP-3, -7, -12, -13, and -14 as well as ADAMTS-5 and -4 can cleave Dcn [34,44]. For Dcn, MMP-14 can generate a non-glycanated isoform similar to those found in aging [38]. ADAMTS proteases and MMP-13 have preferential cleavage regions around the middle of Bgn and Dcn, generating characteristic “half-moon” structure. Some MMP docking and activation can also be influenced by highly sulfated GAGs and their attachment sites [45].

Osteoblasts mostly express MMP-2, -14, ADAMTS-4/5, and TIMP2, while osteocytes express MMP-2, -11, -13, -14, and ADAMTS-4/5 [20,30,31,32]. The increase in MMP-13 expression and decrease in TIMPs have also been associated with osteocyte maturation and mineralization [32]. Osteoclasts express MMP-2 and -9, although they are not generally thought to contribute to changes in proteoglycan turnover within existing mineralized matrix. Intracellularly, PG core proteins can be endocytosed and digested in the lysosome by cathepsins, and the resulting amino acids are then recycled for other cellular functions and protein synthesis. PGs can also be released from the native structure indirectly, via degradation of a binding partner such as collagen or mineral (hydroxyapatite) [46].

GAGs can also be broken down enzymatically (e.g., chondroitinases and lysosomal hydrolases) or non-enzymatically (via chemical or physical forces). With the exception of heparanases, enzymatic digestion of GAGs primarily occurs within lysosomes, as described elsewhere in more detail [40]. Briefly, extracellular GAG fragments or intact proteoglycans are first endocytosed into the cell. Then, large GAG polysaccharides are cleaved into smaller, oligosaccharides by endosomes/lysosomes. Next, sulfatases remove sulfate groups in a stepwise, GAG-specific manner, after which glycosidases subsequently digest the chains into their respective monosaccharide units. The resulting monosaccharides are then recycled by the cell. Lysosomal dysfunction or stress can alternatively result in premature exocytosis or accumulated partially degraded GAG fragments.

To address the non-enzymatic loss mechanisms, GAGs are highly stable molecules due to their strong anionic charge and are therefore most likely to be degraded via mechanical damage or oxidative stress. Mechanical forces are a plausible mechanism contributing to non-enzymatic degradation as bones are constantly mechanically loaded through exercise as well as daily activities like walking. Age-associated damage accumulation and microcracking in bone are well-recognized features of bone, particularly for prominent molecules like collagen [47]. In fact, some studies have demonstrated that nanoscale microcracking increases significantly under fatigue loading when resorption is impaired [47,48]. Furthermore, bone regions beyond cellular reach are less actively regulated and, consequently, remain subject to continuous damage accumulation. Interestingly, microdamage is usually most prominent in the oldest regions of bone (i.e., interstitial tissues), although it is unclear whether this reflects greater cumulative damage over time or the irregular organization and relative weakness of structures like cement lines, which may serve as sites for crack initiation [49,50].

Oxidative stress in the form of reactive oxygen species (ROS) can also lead to the fragmentation of GAGs, with non-sulfated CS and HA being among the most susceptible [42,43]. In contrast, sulfated GAGs such as chondroitin-4-sulfate (C4S) are more resistant to ROS and are even considered to have antioxidant properties. In one study, exposure of SLRPs, Bgn and Dcn to hydroxyl radical flux for 24 h resulted in substantial core-protein degradation, accompanied by partial GAG depolymerization [41].

### 2.2. PG Dynamics in Bone Remodeling

**Figure 1:**
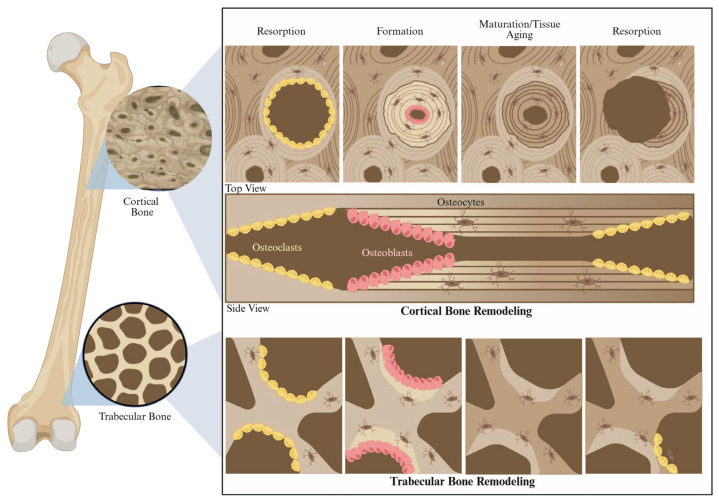
Schematic of the bone remodeling process. Cortical and trabecular bone remodeling begins with resorption of old bone tissue, followed by new bone formation. Created in BioRender. Heath, S. (2026) https://BioRender.com/sscwn4b.

Bone is a living, dynamic tissue constantly undergoing remodeling, either through a multicellular process (the Bone Modeling Unit (BMU)) or an osteocyte-mediated process known as perilacunar canalicular remodeling (PLR). Over time, segments of bone tissue are resorbed and replaced with new cylindrical structural units called secondary osteons in cortical bone. Trabecular bone remodeling follows the same sequence, although the BMU travels along the surface of trabeculae rather than cylindrically tunneling through bone [51]. The BMU represents the principal mechanism of bone turnover and is characterized by: (1) resorption of bone by osteoclasts, (2) formation of new osteoid by osteoblasts, and (3) maturation and maintenance of the tissue, as illustrated in Figure 1. Throughout life, this continuous turnover replaces older tissue with newly formed bone tissue, generating a complex mosaic of new and old tissue throughout the bulk bone. Therefore, bone essentially ages from two interrelated pathways: intrinsic, chronological material (tissue-level) aging and systemic donor-age-associated change in turnover dynamics.

#### 2.2.1. PGs in Bone Formation

**Figure 2:**
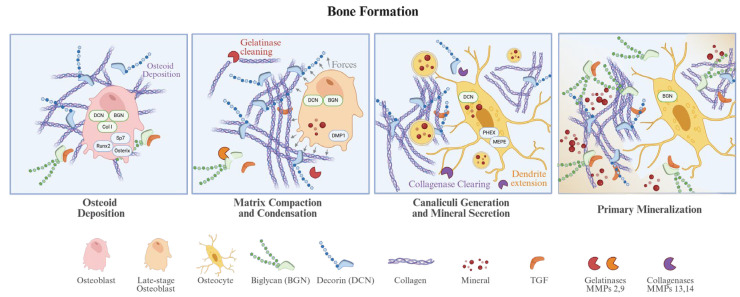
Schematic of the bone formation process during bone remodeling. Bone formation includes osteoid deposition, matrix compaction, mineral secretion, and primary mineralization. Created in BioRender. Heath, S. (2026) https://BioRender.com/9v2ilsf.

Active osteoblasts synthesize and secrete the organic matrix called osteoid (Figure 2). This matrix is primarily composed of type I collagen, alongside fibronectin and PGs, including Bgn and Dcn. In early bone formation, the DS/CS ratio within PGs is higher, since DS is more flexible and can wrap around collagen fibrils to regulate their diameter and lateral alignment [52]. Recent live-cell imaging studies reveal that these fibrils are not static; rather, they are dynamically repositioned as individual cells exert contractile forces to organize the fibers [53]. As the bone nodule begins to form, a subpopulation of osteoblasts differentiates into pre-osteocytes [53]. This transition is marked by upregulation of dentin matrix protein 1 (Dmp1) and E11/podoplanin. The cells progressively adopt a dendritic morphology while expressing non-collagenous proteins like matrix extracellular phosphoglycoprotein) (MEPE) and phosphate-regulating endopeptidase homolog, X-linked (PHEX). Pre-osteocytes may become embedded within the matrix through entrapment by surrounding osteoblasts, migration into forming collagen lacunae, or gradual differentiation in situ [53]. Over the subsequent days, the collagen fibril network undergoes compaction and condensation generated by pre-osteocyte-associated proteinase expression and/or mechanical traction forces generated by the cells.

During mineralization, Bgn (not Dcn) undergoes a functional switch, shifting from DS- to CS-rich forms. In primary bone cell culture models from rats (bone marrow-derived mesenchymal stem cells (BMSCs)) and bone cells derived from alveolar bone explants), mRNA analyses revealed that DS-substituted Bgn is expressed first during phases associated with cell proliferation, declines at early matrix deposition, and subsequently is expressed again in a CS-substituted form at the onset of mineralization [54]. CS-substituted Bgn localizes around the existing collagen scaffold in the extrafibrillar matrix. These CS-rich PGs that are not tightly bound to collagen are thought to play a primary role in regulating mineralization [55]. Due to their anionic nature, these GAGs attract and stabilize hydroxyapatite-associated ions like calcium and generate localized areas of concentrated ions that may serve as nucleation sites. Hydroxyapatite crystals then deposit within and around collagen fibrils. At the same time, GAGs regulate crystal growth to prevent excessive mineralization by acting as regulatory “caps”, ensuring the bone retains toughness and prevents brittleness. This process is further regulated by the balance of inorganic phosphate and pyrophosphate, influenced by proteins like tissue non-specific alkaline phosphatase (TNAP) [56]. Once mineralization begins, previously motile pre-osteocytes become completely immobilized within their mineralized lacunae. At this stage, the cell is considered a mature osteocyte, characterized by the expression of sclerostin (SOST) and FGF23, and is fully integrated into the lacunocanalicular network, where its dendrites act as mechanosensors.

#### 2.2.2. PGs in Tissue Aging and Perilacunar Remodeling

**Figure 3:**
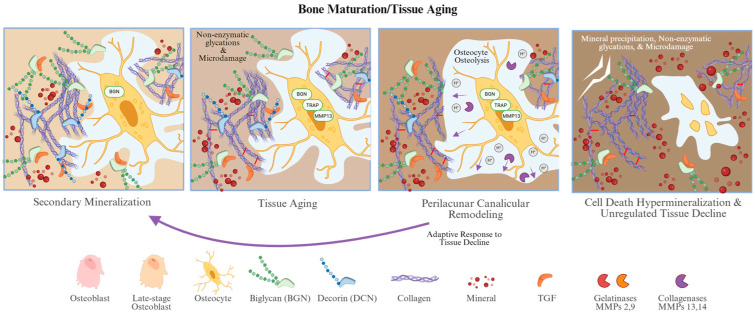
Schematic of the bone maturation process. Bone maturation starts with secondary mineralization. Over the course of decades, the tissue material will age, accumulating non-enzymatic glycations and microdamage. The nearby osteocytes will adaptably respond to this tissue damage by resorbing and remodeling its surroundings in a process called perilacunar canalicular remodeling. The osteocyte regulates bone quality until exhaustion and cell death. If cell death does not trigger osteoclast recruitment and resorption, the bone tissue will continue to decline through cell-free pathways. Created in BioRender. Heath, S. (2026) https://BioRender.com/qczkw31.

Between bone formation and resorption events, the life of local cortical bone tissue is approximately 25–30 years. Trabecular bone turnover is much faster, with local life approximately ~10–15 years [57]. During this aging process, bone undergoes significant compositional changes that directly impact its material properties (Figure 3). Several studies have quantified compositional changes between newly formed bone, and older interstitial tissue in cortical bone. Regarding mineralization, interstitial tissue is significantly more mineralized than osteonal bone [58,59]. Carbonate substitution occurs spontaneously during this process, but not as fast as mineral crystal growth, resulting in a decreased carbonate/phosphate ratio over time [59,60]. Sex-specific trends in mineral quality have also been reported. For instance, the degree of tissue mineralization (Tt. DMB-Al) tends to decrease significantly with age in females, a correlation not consistently observed in males [58]. In terms of the organic matrix, the overall quantity of collagen remains relatively stable across tissue types [61,62], but the quality of collagen declines with age. Specifically, older interstitial tissue accumulates much higher levels of both enzymatic [61] and non-enzymatic crosslinks [61,62,63]. Notably, the distribution of non-collagenous proteins and GAGs is higher in younger bone. Essential non-collagenous proteins like osteopontin and osteocalcin, along with the Dcn, are found in higher concentrations within osteons [62,64]. In contrast, Bgn appears to be more uniformly distributed across tissue ages [64]. Collectively, these findings indicate that as bone transitions from new osteonal to older, interstitial tissue with age, it progressively loses regulatory, structural, and hydrophilic matrix components that support metabolic activity and bone toughness, resulting in a more highly mineralized but also more brittle tissue.

During this tissue-aging period, osteocytes also undergo perilacunar/canalicular remodeling (PLR), where they resorb perilacunar bone matrix and deposit new bone surrounding their lacunae and canaliculi. The lacunar–canalicular network (LCN) facilitates nutrient transport and cell signaling for osteocytes embedded deep within the mineralized matrix. Thus, osteocytes are often thought to play a central role in maintaining bone quality in regions not undergoing BMU-mediated remodeling process. PLR remains an active area of research, and emerging evidence suggests that there are measurable material gradients in the matrix immediately surrounding osteocytes. Kerschnitzki et al. found that ~80% of the mineralized matrix is located within 1.4 µm of the LCN [65]. They further observed that regions with dense osteocyte networks had highly oriented bone mineral, characterized with homogenous and thick mineral crystals that aligned parallel to long axis of osteocyte and perpendicular to canaliculi [65].

#### 2.2.3. PGs and Osteocytes in Bone Resorption or Unregulated Tissue Decline

The lifespan of an osteocyte is around 25 years, similar to that of the surrounding bone matrix. After sufficient mechanical stress or accumulated damage, the osteocytes may undergo apoptosis, causing the adjacent osteocytes to upregulate RANKL, thereby promoting osteoclast recruitment and bone resorption. Furthermore, initiation of bone remodeling requires osteocyte-derived signaling. For example, Rumpner et al. showed that osteoclasts seeded onto cadaveric bone had no response to topological differences such as microdamage in the absence of viable osteocytes [48]. It is speculated that reduced osteocyte density or suppressed osteoclast activity (e.g., during anti-resorptive therapy) may impair proper regulation of the whole bone matrix, resulting in more regions with diminished cellular oversight. Consequently, as these unregulated tissue regions accumulate, the bone microenvironment may undergo significant quantitative and qualitative depletion of PGs and CS GAGs.

## 3. Age-Related Changes in PGs in Bone

**Figure 4:**
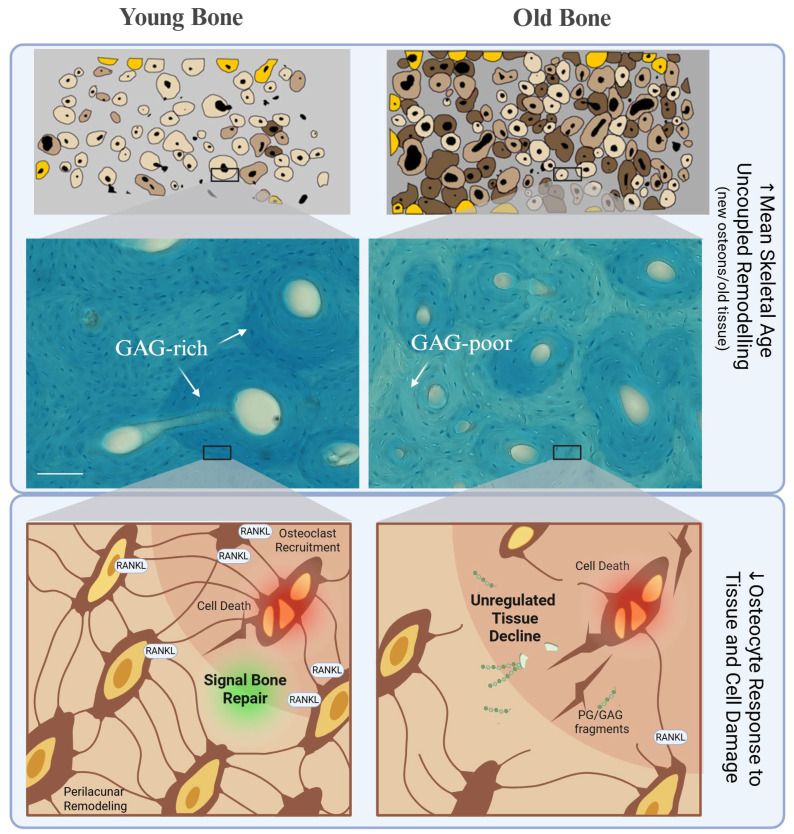
Schematic of major age-related changes in bone. With age, cortical bone has a higher fraction of older interstitial tissue rather than newly formed secondary osteons. Secondary osteons are areas where GAGs are typically present at higher concentration. Over time, tissue aging and reduced osteocyte density may impair cell–cell communication and create regions of matrix that receive less cell interference. Together, the higher proportion of older tissue and reduced osteocyte-mediated maintenance leads to a severe age-related loss of GAGs and reduced bone toughness. Scale is 100 microns. Created in BioRender. Heath, S. (2026) https://BioRender.com/vmydrdo.

During aging, the bone microenvironment experiences both quantitative and qualitative depletion of PGs and CS GAGs. In the hierarchical architecture of bone, these changes likely depend on multiple, interacting factors, including age-related shifts in the proportion of old versus newly made tissue, the distribution of PGs and GAGs within those tissues, as well as changes in cell activity and cell number (Figure 4).

### 3.1. Bulk Changes in GAGs/PGs in Bone

A summary of the diverse age-related changes in PG and GAG expression, ranging from gene downregulation to alterations in bulk tissue content, is provided in Table 3.

#### 3.1.1. PG Levels During Skeletal Development and Maturation

During embryonic development and early childhood, rapid cortical bone formation generates highly organized lamellae sheet structures called primary bone [74]. One study reported that during early development (<15 years of age) CS PGs (CSPGs) and Bgn constitute a major proportion of PGs in human bone [66]. Research on human bone cells demonstrates that juvenile osteoblasts produce PGs with significantly longer GAG chains and higher sulfate content, with synthesis rates reaching three to four times higher than in donors over age 30 [67].

Regarding the quality of these PGs, young matrix possessed a high ratio of Chondroitin-6-Sulfate (C6S) relative to Chondroitin-4-Sulfate (C4S) [54]. This specific sulfation promotes rapid ion diffusion and mineral nucleation, required for skeletal growth [54]. By skeletal maturity (~30 years of age), the synthesis rate of PGs drops significantly, and the total GAG content within the matrix is reduced by approximately half [66].

#### 3.1.2. Age-Related Changes in GAG/PG Content, Type, and Distribution

In adults, GAG content in bulk cortical tissue has been reported to decrease by as much as 17% with aging, while the underlying mechanism behind such loss remains unclear [14]. Another study has reported a 1.5-fold linear increase in iduronic acid substitution in CS chains with age, suggesting a compositional shift toward DS-like structures [66]. Several groups investigating morphological changes in GAGs/PGs have reported age-related reductions in the GAG-containing PG forms of Bgn and Dcn in both bone and cartilage [68,75,76,77,78]. In elderly cartilage, enzymatically deglycosylated BG had 3 prominent bands around 39–45 kDa and 6 smaller fragments ranging from 16 to 35 kDa [78]. Similarly, in intervertebral discs, non-GAG-substituted BG forms were observed at similar sizes of 37 and 41 kDa [68]. Dcn has exhibited fragment sizes of 38, 36, 25, 18, 16, and 14 kDa in elderly cartilage [78], while the non-glycanated forms in intervertebral discs were reported at 43 and 45 kDa [68]. Specifically, sequence analysis of Dcn, found cleavage sites immediately following the serine residue within the GAG attachment motif (N’DEAS/GIG), indicating that age-related processing may occur directly at the GAG substitution site. Another study in cartilage further reported that these non-PG core protein forms increase with age [68].

#### 3.1.3. Systemic Modulators of Bone Matrix Aging

While the local bone microenvironment is the primary site of chronic PG dysregulation, simultaneous age-related shifts in hormonal, metabolic, renal, and skeletal health can further compromise PG levels and bone quality. In women, the most significant driver is the menopausal decline in estrogen, which uncouples bone remodeling by accelerating osteoclast activity. Beyond reducing bone mass, estrogen deficiency alters matrix quality, with studies showing post-menopausal women having lower glycosaminoglycan (GAG) levels than premenopausal counterparts [69]. This degradation of material quality may partially explain why elderly women face higher fracture risks than men, even when controlling for bone mineral density (BMD) [12].

Adding to these hormonal shifts is a rise in oxidative stress. Elderly populations and those with metabolic disorders exhibit elevated reactive oxygen species [79] and markers of oxidative damage [80,81]. Furthermore, when paired with the age-related decline of antioxidant defenses like SOD or glutathione, this high-oxidative environment facilitates the non-enzymatic degradation of extracellular GAGs [81].

Parallel to these metabolic changes, declining renal function further impairs skeletal integrity. Normally, soluble tissue remnants from bone resorption are filtered and excreted by the kidneys. However, age and age-associated pathologies (diabetes, chronic kidney disease) lead to impaired renal clearance, which causes tissue fragments to accumulate [82,83,84]. An increase in these fragments can trigger pro-inflammatory signaling to further accelerate osteoclastogenesis and matrix degradation [85,86,87].

These systemic vulnerabilities are often worsened by localized skeletal conditions, such as previous fractures or joint replacements. With advancing age, this repair process after fracture is further compromised by a blunted inflammatory response and cell recruitment, which significantly delays the deposition of the cartilaginous callus [88]. This prolonged state of impaired healing accelerates the enzymatic degradation of the provisional matrix, likely leading to a premature loss of PGs and GAGs before they can be integrated into the mineralized bone [88]. Additionally, clinical interventions like rigid metallic implants can induce stress shielding. By absorbing mechanical loads, these implants disrupt osteocyte signaling and promotes unregulated matrix maintenance [89]. This localized embrittlement increases the risk of periprosthetic and subchondral insufficiency fractures and can trigger a pro-inflammatory feedback loop, further compromising the bone-implant interface [90].

### 3.2. Age-Related PG Changes in Local Osteocyte Function and Bone Remodeling

#### Osteocyte PG Dysfunction

Both osteocyte numbers and activity are altered with aging, which can have an impact on PG regulation in bone matrix. At the lacunar level, Pei et al. investigated PG turnover rates in the osteocyte pericellular matrices (PCMs) of 15-week and 65-week-old mice using a metabolic labeling approach [70]. While the average intensity of osteocyte PCM was similar with age, the osteocytes in older bone had a more homogenous distribution. While not studied with aging, they noted that conditions increasing PCM turnover (e.g., mechanical loading) also had increased levels of MMP14 [70]. Hagen et al. found that primary osteocytes from 24-month-old mice had reduced PCM formation and mechanosensitivity compared to their 3-month-old counterparts, although pericellular damage repair was faster [73]. The influence of aging on osteoblast PCM and the turnover of the initial PG-rich osteoid matrix remain to be investigated.

Kaya et al. have conducted extensive transcriptomic analyses of mature osteocytes isolated from 2-month-old, 1-year-old, 2-year-old, and 2.5-year-old wild-type mice [71]. Examination of their Supplemental Table S1 identified age-related changes in genes involved in GAG synthesis and regulation [71]. Among PG core proteins, Bgn, Dcn, and Lum were the most expressed PG genes, and all were downregulated with age. Most genes involved in GAG biosynthesis were either slightly downregulated or unchanged with respect to the 2-month-old group. On the other hand, osteocytes from 2-year-old mice had an increase in Mmp-2, -8, and -14 as well as MMP inhibitor Timp-2. Interestingly, osteocytes from 2.5-year-old mice had relatively limited differences in MMP or PG gene expression compared to the 2-month-old mice. Vahidi et al. reported the fraction of individual osteocytes undergoing PLM remodeling was reduced between 5-month-old and 22-month-old mice, accompanied by a decrease in MMP-14-positive osteocytes [72].

Additionally, osteocyte lacunar density and number are reduced both with age [91,92,93] and tissue aging [94]. It is not yet clear whether PLM has a limit in range, although the reported values hover around 10–20 µm from the cell [95,96]. Essentially, this means that the reach of perilacunar remodeling may leave large volumes of bone unregulated to age materially (non-enzymatically) until resorption. This localized cellular failure also contributes to a broader shift in the mean skeletal age of the entire bone.

### 3.3. PGs in Mean Skeletal Aging

With the continuous replacement of old bone by new bone, tissue is intrinsically heterogeneous, exhibiting a local range of tissue ages around an overall mean tissue age. Throughout life, this distribution of tissue ages evolves in response to remodeling dynamics. In 1963, Hattner et al. mathematically suggested that this remodeling process occurs randomly, replacing both primary and older secondary osteons with similar probability [61]. Based on the initial probabilistic assumption, the efficiency for replacing old bone decays exponentially, causing the approximate mean tissue age to increase over time [61]. Additionally, histomorphometric approaches can approximate how the mean tissue age of the bone is changing with donor age. Several studies found that average tissue age increases with donor age, as reflected by the reduced areal fraction ratio of new osteons to older interstitial tissue, in line with previous results reported in the literature [97,98,99]. Other studies have shown that the area fraction of newly formed secondary osteons relative to the total area decreases with donor age [98,99]. However, some studies reported no significant age-related changes in the total number of intact osteons (both secondary and atypical) [100]. The apparent discrepancy may stem from how bone is quantified. Specifically, age-related increases in total osteon population density [97,101,102], can mask a concurrent decline in the relative fraction of newly formed tissue.

Furthermore, while theoretical models are effective in demonstrating the age-related increase in mean tissue age [97], they rely on the assumption of a constant bone turnover rate. The histomorphometric approach is a good snapshot of tissue age but does not account for the age-related changes in remodeling rates. In reality, the average bone turnover rate (~5% per year for cortical bone and ~25% for trabecular bone), leading to complete turnover every 20–25 years [103,104]) is not constant with age. With advancing age, particularly in women, bone turnover becomes uncoupled; there is an increased rate of bone resorption by osteoclasts accompanied by a decreased rate of bone formation by osteoblasts. This uncoupling is critical because it can further amplify the already increasing mean tissue age predicted by the constant-rate model. Table 4 outlines age-related changes associated with bone formation and resorption.

This decrease in bone formation efficiency is especially critical, as it accelerates the accumulation of older, brittle tissue, thus driving the intrinsic loss of bulk bone quality.

## 4. Implications for PG Loss in Bone

PGs and their GAG side chains function as structural scaffolding and regulatory components of the bone matrix. Their roles range from structural organization during development and bone formation to managing mineral density and cell signaling in repair and remodeling of the adult skeleton. Moreover, the specific type of GAG and PG can play unique roles in regulating bone. For example, CS/DS type GAGs tend play a larger structural role on bone matrix, while HS types tend toward mechanical signaling impairments. Table 5 summarizes the skeletal implications of PG dysregulation, detailing how genetic mutations and knockouts that disrupt PG synthesis or assembly result in severe clinical and experimental phenotypes.

### 4.1. Role of PGs and GAGs in Endochondral Ossification

During early development, the physical presence of GAGs dictates the dimensions and mechanical properties of the bone osteoid. Highly negative, long CS chains attract water and generate an internal pressure required to expand the growth plate, resist compression and early ossification. Full knockout models and clinical mutations affecting CS GAGs as well as early-stage GAG synthesis generally have dwarfism phenotypes with a hypocellular, thin proliferative zone that is differentiated and ossified early [110,111,112,113,114,115,128,129,131,132]. Loss of enzymes responsible for linking GAGs to core proteins is even more severe, resulting in severe dwarfism and perinatal lethality. B3GAT3 knockout mice result in embryonic mortality [115,131] and clinical mutations in B3GAT3 phenotypically present multiple bone fractures, shorter stature, and bone dysplasia in infants [110,111,112,113,114,132]. Inhibition of CS/DS GAGs via CSGalNAcT knockout led to postnatal lethality with impaired endochondral ossification and showed malocclusion, general skeletal dysplasia and skin hyperextension, closely resembling Ehlers–Danlos syndrome [128]. Interfering with O-Glycan GAGs (CS, DS, HS, and some KS) via Galnt3 knockout leads to profound mineral imbalances [129]. In the case of Galnt3, this resulted in hyperphosphatemia, tumor calcinosis, and hyperostosis with inhibition of alkaline phosphatase [129]. Interestingly, bone volume (BV/TV) and bone area (BA) were elevated in the Galnt3 knockout, which was more prominent in males [129,130]. Similarly, Bgn knockout models [118,133] and clinical mutations [116,117] also exhibit significant long bone shortening and skeletal dysplasia. Dcn loss had minimal effects on cortical and trabecular bone structure, but impaired local and bulk bone mechanical properties [118]. Furthermore, these two act to partially compensate each other, where Dcn was largely upregulated for Bgn KO while Bgn was moderately upregulated for Dcn KO [118].

### 4.2. Role of PG and GAG Loss in Matrix Quality and Organization

Early-stage PG GAGs like Bgn and Dcn directly interact with collagen, and the GAG attachments can interlock between the fibrils, generating an organized structure. Defects in epimerization (DSE) that converts CS to DS result in altered collagen fibril diameter and irregular shapes for DSE knockout models [127] as well as in conditions like Ehlers–Danlos syndrome [52], although this did not result in an visible bone defect and has not been directly studied in bone to the authors knowledge. Sulfation of CS is also critical for mineralization. In fact, one of the phenotypes of chondroitin 4-O-sulfotransferase-1 knockout models results in impaired mineralization and skeletal development [125].

The PG core proteins show similar effects to loss of CS. In models like the Bgn knockout, there was a loss of regulated collagen-GAG spacing [118]. When Dcn is also absent, as seen in the Bgn/Dcn double knockout, the synergy between these two small leucine-rich PGs (SLRPs) is lost entirely [118]. The resulting matrix is severely disorganized, with irregular collagen fibril diameters that mirror the fragility seen in human Ehlers–Danlos syndrome, creating an exceptionally fragile matrix.

### 4.3. Role of PG and GAG Loss in Mechanical Behavior

**Figure 5:**
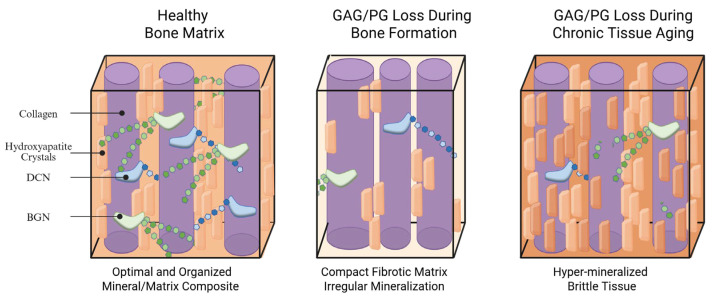
Schematic of bone lamellae ultrastructure revealing structural consequences of PG/GAG depletion during bone formation and chronic tissue aging. Created in BioRender. Heath, S. (2026) https://BioRender.com/xi9yqxm.

The presence of PGs/GAGs during bone formation plays a highly critical role in first regulating collagen fibril structure and quality, then regulating the mineral volume fraction (Figure 5). Mechanically, bone ultrastructure functions as a lamellar composite, with an extrafibrillar hydroxyapatite matrix (analogous to “concrete”) reinforced with mineralized collagen fibrils (analogous to “steel rebar”). The elastic behavior of lamellar composite can be largely assessed by the rule of mixture, whereas plastic behavior and toughening mechanisms are strongly influenced by interfacial interactions [134]. Thus, the presence of GAGs/PGs during this time can govern overall bone strength and toughness. Although long-term effects have not been fully studied, there is evidence that removal of glycosaminoglycans (GAGs) leads to increased mineralization of bone and ligament tissues [135,136], suggesting that GAG loss can increase overall bone stiffness via mineralization.

Beyond that, GAGs can directly contribute to bone plastic energy dissipation via retaining bound water. 2D in silico simulations of bone ultrastructure, revealed that plastic behavior of bone largely results from residual deformation of the hydrated interfaces between collagen and hydroxyapatite [137], which are zones rich in GAGs. Specifically, the plastic energy from mineralized collagen arises from the interface with the extrafibrillar matrix, while the plastic energy from the extrafibrillar matrix stems from the interfacial behavior between hydroxyapatite platelets [137]. In fact, enzymatic digestion of GAGs resulted in loss of energy dissipation, primarily in the extrafibrillar matrix and increase in elastic modulus for both phases [138]. It was speculated that removal of GAGs caused a loss of bound water, that ultimately led to shrinking and hardening of the tissue.

While the effect in bone was strongest in the GAG-rich mineral phase, evidence in soft tissues like tendons suggest that intrafibrillar GAGs can also influence the collagen phase, although this effect is variable between studies. GAG removal in the Achillis tendon resulted in similar initial mechanical behavior but had higher microdamage accumulation overtime [139]. The authors attributed this effect to GAG-mediated regulation of load transfer across adjacent fibrils, thereby helping resist fiber damage and retain integrity [139]. An AFM study found GAG depletion lowered collagen fibril stiffness and indentation modulus [140]. Other studies found that loss of interfibrillar GAGs had limited effects on viscoelastic or structural properties [140,141,142]. These experimental variations may be attributed to differences in hydration status, GAG removal, mechanical assessment, length scale, tissue age, and tissue type. Therefore, the significance of GAGs within intrafibrillar collagen is yet to be fully understood. Overall, these damage models help establish the direct role of GAGs on bone mechanical behavior; however, the direct role of the core proteins Bgn and Dcn have yet to be experimentally assessed independently from knockout models (where bone formation and volume are concurrently compromised). Studies with inducible knockout models, targeted damage models, or in silico approaches may be able to provide such clarifications in the future.

### 4.4. Functional Role of PGs and GAGs Loss in Cell Signaling

**Figure 6:**
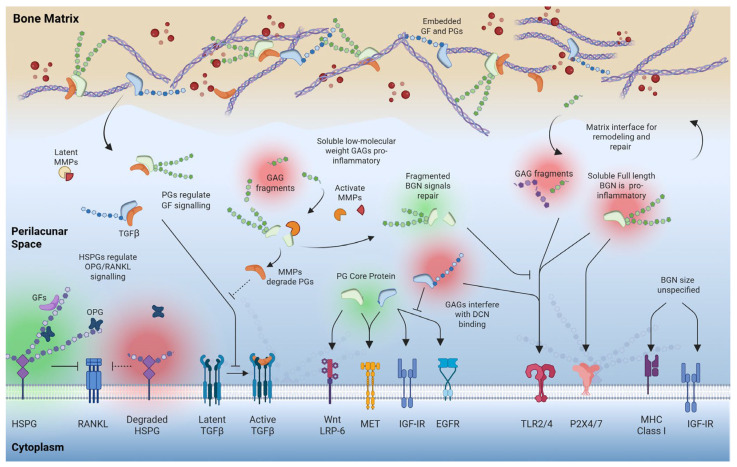
Functional role of intact and fragmented forms of PGs/GAGs on cell signaling. Created in BioRender. Heath, S. (2026) https://BioRender.com/kgjdhe7.

Extracellular PGs function as more than structural scaffolds; they also regulate several cell signaling pathways (Figure 6). HS PGs on the cell surface and within the pericellular matrix act as co-receptors and modulators of growth factor signaling. HS plays a key role in regulating the RANKL/OPG axis, acting as a direct ligand for both OPG and RANKL [143,144,145], thereby modulating osteoclastogenesis and bone resorption. In fact, when this interaction is disrupted like with heparinase treatment, cells exhibit an elevated RANKL/OPG ratio to increase bone turnover [146].

The importance of HS in bone formation is further highlighted by the Ext1 knockout model (HS co-polymerase). The disruption of HS synthesis does not mirror the gross skeletal abnormalities observed in CS knockouts, but instead specifically alters cellular kinetics. In these models, loss of HS leads to increased chondrocyte proliferation and a significant delay in differentiation, demonstrating that HS is a key regulator of the rate of skeletal maturation [120]. This suggests that while CS contributes predominantly to bulk structural integrity, HS provides the temporal regulatory control over cellular development.

Aside from HS, SLRPs like Bgn and Dcn can also act as ligands to connect bone quality with cell response. Dcn is a ligand for several receptor tyrosine kinases and signaling proteins like Met, EGFR, VEGFR2, TGF-beta, as well as innate immune receptors TLR2, and TLR4 [147]. Similarly, Bgn interacts with TLR2, TLR4, P2X4/P2X7, MHC Class 1, IGF-IR and Wnt co-receptor LRP6 [147,148]. SLRP-mediated signaling appears to be important, where Bgn and Dcn KO mice have severely impaired ERK and p38 MAPK signaling pathways [118]. This suggests that without these PGs, cells are less capable of sensing anabolic cues and stress-repair signaling responses.

### 4.5. Functional Role of Catabolic Products of PG and GAG Loss in Matrix Regulation

In the adult skeleton, PGs are also important mediators of the catabolic-to-anabolic transition during bone repair. During catabolic events like tissue aging, mechanical injury, or chronic inflammation, PGs can become damaged or degraded. Not only are PG fragments increasingly being used as a biomarker for pathogenic extracellular matrix remodeling [46], but these breakdown products can also act as powerful signaling molecules to regulate further tissue degradation or remodeling.

The biological impact of these PG fragments depends on their molecular composition. For example, in chondrocytes, CS fragments can stimulate production of catabolic enzymes such as MMP-13 to create a loop of matrix destruction [149]. Similarly, soluble low molecular weight HS and HA fragments, but not full HS chains, can also bind to Toll-like receptors (TLR) and promote upregulation of cytokines [150,151]. Conversely, DS fragments released during injury have been found to activate FGF signaling, suggesting a role in initiating regenerative responses [85,86].

Fragments of the SLRP core proteins play a distinct role in cell activity compared to their full and glycosylated counterparts. While full-length Bgn can interact with inflammatory receptors (TLRs), Bgn fragments and the Bgn core protein had a very limited effect on the p38-proinflammatory response [87] and are even associated with ECM remodeling. One study found that Bgn fragments were higher in healthy discs compared to those with intervertebral disc degeneration [152]. In fact, they reported that full length Bgn increased expression of FGF-17 and the ADAM1B metalloproteinase, while fragmented Bgn upregulated ECM organization pathways and downregulated inflammation and lipid metabolism [152]. In other studies, Bgn fragments were found to be elevated in synovial fluid under active remodeling conditions, such as horses undergoing mechanical loading and in those with subchondral bone sclerosis or chip fractures [153]. Dcn fragments follow a similar principle of specialized function. Although Dcn fragments remain under-investigated as independent ligands, the Dcn core protein interacts directly with EGFR to activate ERK signaling [154]. Interestingly, the GAG chain interfered with binding, suggesting a pro-remodeling role in ECM organization [154].

Essentially, PG quality may thus serve as a detector (the “fire alarms,” if you will) of total bone quality, with soluble GAG remnants and GAG-containing PGs facilitating further matrix destruction, while PG core proteins may signal repair. However, there is limited understanding of reversibility or the regulatory mechanisms that shut down PG catabolic signaling, so PG loss/fragmentation is consequently linked to several positive-feedback loop disorders like diabetes and aging. Breaking these cycles requires targeted interventions, ranging from pharmacological replenishment to the stimulation of cellular remodeling.

## 5. Potential Interventions for Age-Related GAG/PG Loss

This section explores strategies to mitigate PG and GAG depletion, focusing on restoring the matrix’s material quality and silencing the catabolic feedback loops associated with skeletal aging. While these approaches represent promising avenues for future clinical agents, they must be carefully balanced to avoid disrupting essential physiological remodeling.

### 5.1. Pharmacological and Biological Replenishment

Direct replacement with GAG supplements and synthetic PGs offers a potential pathway to improve mechanical behavior and cell metabolism. In experimental models, direct intradermal supplementation of chondroitin sulfate (CS) improved bone GAG levels and bound water in wild-type mice, while also inhibiting osteoclast activation [15]. However, clinicians must consider a reintegration barrier in aged bone: hyper-mineralized extrafibrillar matrix and reduced lacunocanalicular porosity likely limit the ability of exogenous GAGs to penetrate deep into older interstitial bone to re-bind with bone matrix. To overcome this, administering GAGs during windows of high turnover, such as following a fracture or in early-stage osteopenia, may allow for better incorporation into the newly forming mineralizing architecture [155].

Additionally, biological replenishment via gene therapy, such as GlcAT-I or SLRP (Bgn/Osteoadherin) overexpression, has shown success in enhancing GAG synthesis and promoting osteogenic differentiation in vitro [156,157,158].

### 5.2. Osteoblast and Osteocyte Anabolic Stimulation and Turnover

As the mean tissue age of cortical bone increases, the matrix becomes less regulated and more materially compromised. Promoting new bone formation is a critical clinical goal to balance remodeling and restore GAG content. While anti-resorptive drugs (e.g., bisphosphonates) are common, anabolic treatments remain limited. Teriparatide (PTH) has shown the ability to improve GAG levels in post-menopausal women, though levels remained below those of healthy premenopausal controls [69]. New drugs like romosozumab are also considered both anabolic and antiresorptive by blocking sclerostin, although its effect on GAGs is not established.

Beyond bulk turnover, stimulating osteocyte perilacunar remodeling (PLR) through PTH treatment or mechanical loading may rescue older, poor-quality bone [70,73,159,160,161]. These stimuli upregulate MMP-14, which is essential for the healthy turnover of the pericellular matrix (PCM) and the removal of aged, brittle tissue.

### 5.3. Diagnostic Stratification and Inhibiting Catabolic Signaling

A major challenge for clinicians is identifying patients at high risk for fracture before failure occurs. While research strongly links GAG depletion to reduced bone toughness, there is currently no clinically available urine or serum test specifically used to predict fracture risk via these molecules. To bridge this gap, Magnetic Resonance Imaging (MRI) is being investigated as a non-invasive diagnostic tool to measure bound water as an indirect biomarker for GAG-related bone fragility [162,163]. Furthermore, identifying specific circulating GAG fragments or PG remnants (DAMPs) in blood or urine may allow for future risk stratification.

Therapeutically, breaking the positive-feedback loop where GAG fragments trigger TLR-mediated inflammation could preserve matrix integrity. Breaking the positive-feedback loop where GAG fragments (DAMPs) trigger TLR-mediated inflammation may improve bone quality. Small-molecule inhibitors that specifically block the binding site for PG-derived DAMPs on TLR4 may be suitable, but they need to be carefully designed so as not to completely shut down innate immunity. Developing monoclonal antibodies that neutralize defined fragments of Bgn or Dcn and GAG chains before they can bind to macrophages or osteocytes may be a more targeted approach.

Furthermore, another strategy is to proactively block the enzymes responsible for GAG/PG cleavage (MMP-13) to preserve the existing material quality or to administer synthetic TIMPs to restore the natural balance between matrix synthesis and decay. Clinicians must approach enzyme inhibition with caution, as many MMPs are required for housekeeping and healthy remodeling. Prolonged inhibition could inadvertently increase the fraction of old, poor-quality bone, mirroring the risks associated with long-term anti-resorptive therapy.

## 6. Conclusions

The traditional clinical focus on BMD accounts for only half of the variance in fragility fractures, leaving a critical gap in our understanding of skeletal aging. This review highlights that the depletion of the non-collagenous organic matrix, specifically PGs and GAGs, is a primary driver of age-related bone brittleness. As bone turnover becomes uncoupled with age, the accumulation of older interstitial tissue with lower GAGs and reduced osteocyte regulation results in a “dead zone” of unregulated matrix that lacks the hydrophilic and regulatory benefits of fresh PGs. While pharmacological interventions such as PTH treatment and GAG supplementation show promise in restoring these levels, their efficacy is likely limited by the physical barriers of a hyper-mineralized aging matrix.

Ultimately, shifting the therapeutic paradigm from mass preservation to material quality restoration is essential for addressing the rising global burden of fragility fractures. Achieving this requires a rigorous multidisciplinary effort; the complexity of the bone matrix necessitates that basic scientists, clinicians, and engineers collaborate directly to bridge the gap between benchtop material science and orthopedic practice. Future research must prioritize (1) anabolic approaches to increase the fraction of newer, better-quality bone; (2) targeting the lacunocanalicular system to improve existing, older bone; and (3) implementing chemical and physical modifications and supplements to rescue regions of unregulated bone. By integrating diverse expertise, the field can accelerate the development of targeted tools that address the intrinsic root causes of age-related fragility.

## Figures and Tables

**Table 1:** Major genes encoding enzymes and regulators of proteoglycan synthesis and modifications.

Category	Specific Genes/Enzymes	Major Function	Ref
Proteoglycan Core Proteins	BGN, DCN, ASPN, FMOD, LUM	Major bone matrix PG core proteins (e.g., Bgn, Dcn, asporin, FMOD, lumican (LUM)	[16]
Synthesis of GAG Backbone (linkage region and polymerization)	XYLT1, XYLT2, B4GALT7, FAM20B, B3GALT6, B3GAT3, PXYLP1 *, EXT1/2, EXTL3, CSGALNACT1/2, CHSY1/3, CHPF, CHPF2 *	Initiates GAG attachment to Serine residues on the core protein and supports chain elongation	[21,22,23]
CS/DS Post-Translational Modifications	CHST11, CHST12 *, CHST13 *, CHST14, UST, CHST3, CHST15, DSE, DSEL	Sulfates CS chains and supports epimerization to dermatan sulfate (DS)	[21,22,23,24]
HS Post-Translational Modifications	NDST1-4, GLCE, HS2ST1, HS6ST1-3, HS3ST1-6, SULF1, SULF2	Modifies HS (e.g., on syndecans and perlecan	[21,22,23,24]
Regulators & Metabolic Support	PAPSS1, PAPSS2, CANT1 *, IMPAD1, GOLPH3, SPPL3 *	Provides sulfate donor supply and general support for GAG	[21]
Transporters	SLC35B2, SLC35B3, SLC35B4, SLC35A2	Transports nucleotide-sugar and sulfate-related precursors required for PG/GAG biosynthesis	[21]

Note: Associated but under-characterized genes are denoted with *.

**Table 2:** Major Catabolic Factors for PG Breakdown. High expression level in osteoblasts (OB), osteocytes (OCY), and osteoclasts (OC) corresponds to “+++” while no expression is denoted by “-”.

Class	Factor	Specific PG/Core Target	OB	OCY	OC	Cleavage	Location	Ref
Enzymatic	BMP-1	Dcn, Bgn	+++	++	++	Converts pro-forms into mature Bgn and Dcn via N-terminal cleavage	Extracellular matrix	[27,28,29]
	MMP-2	Bgn	+	+++	+++	Multiple cleavage sites reported	Extracellular matrix	[20,30,31,32]
	MMP-3	Bgn, Dcn	-	+	++	Activates other MMPs; cleaves near N and C-termini	Extracellular matrix	[33,34,35,36]
	MMP-7	Dcn	++	-	++	Multiple cleavage sites reported	Extracellular matrix	[20,30,31,32]
	MMP-9	Bgn	++	+	+++	Multiple cleavage sites reported	Extracellular matrix	[20,30,31,32]
	MMP-12	Bgn, Dcn	+++	+	++	Multiple cleavage sites reported	Extracellular matrix	[20,30,31,32]
	MMP-13	Bgn, Dcn	+++	++	+	Several cleavage sites reported	Extracellular matrix	[20,32,37]
	MMP-14	Bgn, Dcn	++	+++	+	Membrane-tethered; activates other MMPs and cleaves SLRPs (including N-terminal regions)	Cell Membrane	[37,38]
	ADAMTS-4/5	Bgn, Dcn	+	+++	+	Cleaves within the core region resulting in a “half-moon” structure of SLRPs	Extracellular matrix	[39]
	Heparinase	HS	+	++	++	Depolymerizes HS	Extracellular matrix	[40]
	Chondroitinase	CS	-	-	-	Depolymerizes CS	Bacterial systems (experimental)	[40]
	Hyaluronidase	HA	+	++	+++	Depolymerizes HA	Lysosome/ extracellular	[40]
Non-Enzymatic	Reactive Oxygen Species (ROS)	Nonspecific	+	++	++	Oxidative fragmentation of GAG chains (often preferential for less-sulfated regions), and core protein damage	Extracellular matrix	[41,42,43]
	Mechanical Damage	Nonspecific	+	+++	-	Mechanical loading-associated fragmentation of GAG chains and proteolysis with damage accumulation	Extracellular matrix	

**Table 3:** Summary of age-related changes in bone PGs and GAGs across species.

Species	Study Focus/Condition	Age Range/Group	Observed Change/Phenotype	Ref
Human	Skeletal Development	Juvenile vs. Skeletal Maturity (~30 years)	Total GAG amount reduced by ~50% by skeletal maturity. CS PGs and Bgn are the major proportion in early development.	[66]
Human	Juvenile Synthesis	<15 years	Juvenile osteoblasts produce PGs with longer GAG chains and synthesis rates 3–4x higher than donors > 30 years.	[67]
Human	Bone Sulfation	Juvenile vs. Adult	Young matrix possesses a high ratio of Chondroitin-6-Sulfate (C6S) relative to Chondroitin-4-Sulfate (C4S).	[54]
Human	Iduronic Substitution	Fetal to 60 years	1.5-fold linear increase in iduronic acid substitution in CS chains with age (transition to DS-like).	[66]
Human	Bulk Cortical Bone	Young vs. Mid-Aged vs. Elderly	GAG content decreases by up to 17% between young (avg. 24 years) and elderly (avg. 73 years); associated with loss of bound water and toughness.	[14]
Human	Tissue Aging	Adults	Decline in Osteopontin, Osteocalcin, and Decorin (Dcn) in older interstitial tissue vs. younger osteons.	[64]
Human	Articular Cartilage	Elderly	Elevated fragmentation of Dcn (14–38 kDa) and Bgn (16–45 kDa) compared to normal controls.	[68]
Human	Intervertebral Discs	Healthy vs. Degenerate	Healthy discs have higher Bgn fragments; degenerate discs have full-length Bgn that upregulates FGF-17.	[68]
Human	Menopause (Iliac Crest)	Pre- vs. Post-menopausal	Post-menopausal women (avg. 70 years) have significantly lower GAG levels than pre-menopausal (avg. 40 years).	[69]
Human	Bone Marrow (Lumican)	Elderly (Hip Fracture)	Lumican 16.9% lower in fracture patients; low LUM correlates with decreased bone mass.	[37]
Mouse	Pericellular Matrix (PCM)	15-week vs. 65-week	Older bone shows more homogenous PG distribution in the osteocyte PCM.	[70]
Mouse	Osteocyte Gene Expression	2-month vs. 1, 2, and 2.5-year	Downregulation of PG core proteins (Bgn, Dcn, Lum). Upregulation of Mmp-2, -8, -14 at 2 years.	[71]
Mouse	Lacunar–Canalicular Turnover	5-month vs. 22-month	Reduced fraction of osteocytes undergoing PLM and fewer MMP-14-positive cells.	[72]
Mouse	Osteocyte Mechanosensing	3-month vs. 24-month	Primary osteocytes show reduced PCM formation and reduced mechanosensitivity.	[73]

**Table 4:** Age-related Changes to Bone Remodeling Properties. Increases and decreases are denoted by ↑ and ↓ respectively.

Bone Remodeling Properties		Change with Age	Ref
Bone Formation Rate	Bone Formation (% new tissue volume/year)	70% ↑ serum BAP 50% ↑ serum BGP 100% ↑ BFR-v	[105]
	Bone formation markers (osteocalcin & propeptide type 1 procollagen)	↓	[106,107]
	Osteoid Volume	↑	[105]
	Mineral Apposition Rate	↑	[105,108]
	Osteoblast Number	No change in number between 7 and 21 month old mice	[108]
Bone Resorption Rate	Bone resorption remnants (C- and N-Terminal Telopeptides of Type I Collagen, Pyridinolines)	↓ after 30 years	[107]
	Osteoclast Activity (secreted Tartrate-resistant acid phosphatase)	Highest before 30 years	[109]
	Osteoclast Number	↑numberbetween 7 and 21 month old mice	[108]

**Table 5:** Skeletal Implications of PG Deficiency: Impact of Synthesis and Assembly Defects on Bone Integrity.

Gene	Species	Genotype/Defect	Skeletal Phenotype	Ref
B3GAT3	Human	Homozygous mutation	Linkeropathy. Multiple fractures, severe osteopenia, short stature, and radio-ulnar synostosis.	[110]
	Human	Faulty initiation of PG synthesis	Joint dislocations, short stature, and cardiac defects.	[111]
	Human	Novel mutation (Nias/Indonesia)	Skeletal dysplasia in a consanguineous clan.	[112]
	Human	Second family report	Skeletal dysplasia, global developmental delay, and multiple congenital anomalies in a 5-year-old.	[113]
	Human	Unique mutation	Craniosynostosis and bone fragility.	[114]
	Mouse	B3GAT3 knockout	Embryonic mortality; impairment of embryonic cell division	[115]
BGN	Human	Loss-of-function (X-linked)	Meester-Loeys syndrome; mild skeletal dysplasia, pectus deformities, and flat vertebral bodies.	[116,117]
	Mouse	Bgn knockout	Significant long bone shortening, skeletal dysplasia, and loss of regulated collagen-GAG spacing	[15]
DCN	Mouse	Dcn knockout	Minimal effect on bone structure but impaired local and bulk mechanical properties	[118]
BGN/DCN	Mouse	Bgn/Dcn double knockout	Severely disorganized matrix and irregular collagen fibril diameters; extreme matrix fragility	[118]
EXT	Human	EXT1/2 deficiency	Hereditary Multiple Exostoses; multiple bony outgrowths (osteochondromas), short stature, and irregular limb length.	[119]
	Mouse	Ext1 knockout	Increased chondrocyte proliferation and significant delay in skeletal maturation/differentiation	[120]
B4GALT7	Human	B4GALT7 deficiency	Larsen-like Syndrome; Short long bones, joint dislocations or laxity, and scoliosis.	[121]
CANT1	Human	Homozygous or compound heterozygous mutations	Desbuquois Dysplasia Type 1. Lethal dwarfism, “Swedish key” or “monkey wrench” proximal femur.	[122]
XYLT1	Human	Homozygous loss-of-function mutations	Desbuquois Dysplasia Type 2. Severe short stature, joint laxity, and advanced carpal ossification without characteristic hand anomalies.	[123]
CHST	Human	CHST14 deficiency	mcEDS-CHST14. Progressive foot/ankle deformities, recurrent joint dislocations (80% by age 10), and kyphoscoliosis.	[124]
	Mouse	CHST11 knockout	Impaired mineralization and skeletal development	[125]
DSE	Human	DSE deficiency	mcEDS-DSE. Similar to mcEDS-CHST14 but often with a lower symptom burden; includes progressive clubfoot and spinal deformities.	[126]
	Mouse	DSE knockout	Altered collagen fibril diameter and irregular shapes	[127]
CSGALNACT	Mouse	CSGalNACT knockout	Postnatal lethality, impaired endochondral ossification, malocclusion, and skin hyperextension	[128]
GALNT3	Mouse	Galnt3 knockout	Hyperphosphatemia, tumor calcinosis, and hyperostosis; elevated bone volume (more prominent in males)	[129,130]

## Data Availability

No new data were created or analyzed in this study.

## Abbreviations

The following abbreviations are used in this manuscript:

AGEs	Advanced Glycation End-products
ADAMTS	A Disintegrin and Metalloproteinase with Thrombospondin Motifs
BAP	Bone-specific Alkaline Phosphatase
BFR-v	Bone Formation Rate (per bone volume)
Bgn/BGN	Biglycan
BGP	Bone Gla-Protein (Osteocalcin)
BMD	Bone Mineral Density
BMSCs	Bone Marrow-derived Mesenchymal Stem Cells
BRU/BMU	Bone Remodeling Unit/Bone Multicellular Unit
CS	Chondroitin Sulfate
DAMPs	Damage-Associated Molecular Patterns
Dcn/DCN	Decorin
Dmp1	Dentin Matrix Protein 1
DS	Dermatan Sulfate
DXA	Dual-energy X-ray Absorptiometry
ECM	Extracellular Matrix
EFM	Extrafibrillar Matrix
EGFR	Epidermal Growth Factor Receptor
ERK	Extracellular Signal-Regulated Kinase
FGF	Fibroblast Growth Factor
FMOD	Fibromodulin
GAGs	Glycosaminoglycans
HA	Hyaluronic Acid
HS	Heparan Sulfate
IGF-IR	Insulin-like Growth Factor I Receptor
KS	Keratan Sulfate
LCN	Lacunocanalicular Network
LRP6	Low-density Lipoprotein Receptor-related Protein 6
LUM	Lumican
MAPK	Mitogen-Activated Protein Kinase
MEPE	Matrix Extracellular Phosphoglycoprotein
MMP	Matrix Metalloproteinase
OB	Osteoblast
OC	Osteoclast
OCY	Osteocyte
OPG	Osteoprotegerin
PCM	Pericellular Matrix
PGs	Proteoglycans
PHEX	Phosphate-regulating Endopeptidase Homolog, X-linked
PLR/PLM	Perilacunar Remodeling/Perilacunar Mineralization
PTH	Parathyroid Hormone
RANKL	Receptor Activator of Nuclear Factor Kappa-B Ligand
ROS	Reactive Oxygen Species
SLRPs	Small Leucine-Rich Proteoglycans
TIMP	Tissue Inhibitor of Metalloproteinases
TLR	Toll-Like Receptor
TNAP	Tissue Non-specific Alkaline Phosphatase
